# Designing Futuristic Telemedicine Using Artificial Intelligence and Robotics in the COVID-19 Era

**DOI:** 10.3389/fpubh.2020.556789

**Published:** 2020-11-02

**Authors:** Sonu Bhaskar, Sian Bradley, Sateesh Sakhamuri, Sebastian Moguilner, Vijay Kumar Chattu, Shawna Pandya, Starr Schroeder, Daniel Ray, Maciej Banach

**Affiliations:** ^1^Pandemic Health System REsilience PROGRAM (REPROGRAM) Consortium, REPROGRAM Telemedicine Study Group, Sydney, NSW, Australia; ^2^Neurovascular Imaging Laboratory & NSW Brain Clot Bank, Department of Neurology, Liverpool Hospital and South Western Sydney Local Health District, Ingham Institute for Applied Medical Research, The University of New South Wales, Sydney, NSW, Australia; ^3^The University of New South Wales (UNSW) Medicine Sydney, South West Sydney Clinical School, Sydney, NSW, Australia; ^4^The University of the West Indies, St. Augustine, Trinidad and Tobago; ^5^Global Brain Health Institute, Trinity College Dublin, Dublin, Ireland; ^6^Department of Medicine, St. Michael's Hospital, University of Toronto, Toronto, ON, Canada; ^7^Alberta Health Services and Project PoSSUM, University of Alberta, Edmonton, AB, Canada; ^8^Penn Medicine Lancaster General Hospital and Project PoSSUM, Lancaster, PA, United States; ^9^Farr Institute of Health Informatics, University College London (UCL) & NHS Foundation Trust, Birmingham, United Kingdom; ^10^Polish Mother's Memorial Hospital Research Institute (PMMHRI) in Lodz, Cardiovascular Research Centre, University of Zielona Gora, Zielona Gora, Poland; ^11^Department of Hypertension, Medical University of Lodz, Łódź, Poland

**Keywords:** telehealth, digital medicine, pandemic (COVID-19), robotics, telemedicine, artificial intelligence, coronavirus disease 2019 (COVID-19)

## Abstract

Technological innovations such as artificial intelligence and robotics may be of potential use in telemedicine and in building capacity to respond to future pandemics beyond the current COVID-19 era. Our international consortium of interdisciplinary experts in clinical medicine, health policy, and telemedicine have identified gaps in uptake and implementation of telemedicine or telehealth across geographics and medical specialties. This paper discusses various artificial intelligence and robotics-assisted telemedicine or telehealth applications during COVID-19 and presents an alternative artificial intelligence assisted telemedicine framework to accelerate the rapid deployment of telemedicine and improve access to quality and cost-effective healthcare. We postulate that the artificial intelligence assisted telemedicine framework would be indispensable in creating futuristic and resilient health systems that can support communities amidst pandemics.

## Introduction

Telemedicine or telehealth is the use of medical information to improve patient's health (1, 2). Reorganization in healthcare delivery, financing, and advancement in electronic health records and clinical decision support systems can accelerate the telehealth adoption into healthcare delivery (2). In this context, artificial intelligence (AI) and robotic technologies can play an important role in the use and delivery of telemedicine during coronavirus disease (COVID-19) and the post-pandemic world (3–10). Our previous two reports have identified gaps in telemedicine across geographics and medical specialties (11, 12). The current paper is a call for the integration of AI, robotics, and telemedicine with an organizational framework powered by AI to accelerate healthcare delivery and improve access to healthcare in the context of public health preparedness and response during outbreaks or public health emergencies such as COVID-19.

## Artificial Intelligence Assisted Telemedicine

Diagnosis is a multidisciplinary process that may involve multimodal testing such as clinical, imaging, blood, and genetic markers. Moreover, discipline-specific testing such as neuropsychological tests may be needed, for example, to obtain comprehensive mental health assessments (13). In a telemedicine framework, some of these testing may be unavailable, while others may be cost-prohibitive. To address this complex multivariate problem in finding an optimal diagnostic protocol, innovative data-driven artificial intelligence (AI) algorithms may offer a solution by applying machine learning to large datasets of disease populations (14, 15). These models can learn directly from the data without any prior statistical modeling, thus producing more objective results while focusing on prediction generalizability for diagnostic purposes on diverse populations. Since the COVID-19 outbreak, international efforts toward COVID-19 forecasting, prevention and treatment are underway using data-driven tools and pooled datasets (16). Moreover, the ML model features an important analysis that enables the search for more cost-effective protocols (17). Unlike traditional statistical hypothesis testing, data-driven computational approaches can test for synergistic variable combinations and redundant feature elimination enabling more effective diagnosis under the specific constraints of telemedicine (18).

Analysis by Collier et al. found that the use of AI applications could result in ~$150 billion in saved healthcare costs annually by 2026 in the United States (19). According to Wahl et al., the ubiquitous use of smartphones, combined with growing investments in supporting technologies (e.g., mHealth, electronic medical record (EMR), and cloud computing), provide ample opportunities to use AI applications to improve public health outcomes in low-income country settings (20). Rapidly increased usage of electronic gadgets accelerates digital shifts in healthcare that appear to become essential in sharing information between and within medical workers and patients (21).

AI exhibits clear advantages over humans in analytical reasoning and problem solving (especially when large amounts of data are involved) and can effectively address the limitations of human function (22). However, the use of AI in healthcare must consider or mitigate the potential loss of vital physician skills if AI is over-utilized (17). Furthermore, rigid algorithm protocols and decision-making trees are subject to the consequences of the inability of AI to fully take in and interpret contextual information or delineate between relevant vs. non-relevant informational input even when employing deep machine learning (23). Contingencies are the norm in healthcare, and the human skill required to navigate and manage this off-nominal, or unpredictable situations must be carefully weighed against the advantages of using AI technology (23–28). User interface and data input methods are critical as voice recognition and interpretation is a major challenge of AI utilization (29). Generalized challenges to utilizing AI in healthcare are created by the fact that many cares and treatment decisions, especially in emergent and time-restricted scenarios hinge on human thought processes that occur in the subconscious, such as intuition, insight, subjective evaluation, and the analyzation of ambiguous or qualitative data (30). Jarrahi illustrates the benefit of the symbiotic use of AI combined with a human in the loop by showing the statistically significant reduction of error (85%) in detecting cancer in images of lymph nodes when compared to AI or human interpretation alone (30).

Telemedicine has provided a critical patient continuity pathway in times of disruption in health service during COVID-19 (10–12). This also helps protect healthcare facilities and minimize risks to health workers during pandemics, which/who are increasingly under pressure (31–33). Telemedicine has the potential to minimize the economic impact on society and healthcare services. Quarantined doctors can provide these services in medical institutions with remote access or care via telecommunications directly to the consumer, freeing other doctors to provide immediate assistance to more needy patients. Teleconsultations allow doctors to evaluate patients, detect signs of infection, and quickly and easily document patients who might be at higher risk of illness (34). Telemedicine solutions work promptly, filling out insurance documents, which allows doctors to devote more time to treating patients. Clinics are expanding their telehealth services to screen patients for COVID-19, which is critical to identifying patients and speeding up medical care, as well as limiting public exposure (11, 32). Bundling of various specialties such as teleradiology, tele-oncology, and telepathology is another area of crucial consideration to facilitate comprehensive management (35).

Further upstream in the telemedical framework, it is important to consider the integration of educational models for physicians and trainees, and particularly how these technologies may address the issues of connecting distributed learners (particularly in the era of a pandemic) (36), and providing high-quality educating, training, and just-in-time training, in a way that is trusted, safe, and replicates the quality of “in-person” training, particularly when it comes to surgical procedures (37, 38). Immersive technologies such as virtual reality and augmented reality could enhance capabilities for collaboration, both amongst learners and physicians in practice (39). Lastly, as has been mentioned elsewhere in this paper, immersive and telemedical technologies, whether used for consultations, treatments, and diagnostics, or education, must be resilient and capable of maintaining at least some degree of functionality even in the absence of internet or network connectivity (40). This is relevant to areas where broadband internet access is poor which limits the effective implementation of these technologies (41).

## Robotics Assisted Telemedicine

Robotics is a promising cutting-edge tool in telemedicine that has potential applications in transforming physical exam and clinical care, as well as monitoring patients in remote conditions (42, 43). Such experience with the Ebola outbreak and with the COVID-19 pandemic has revealed a wide range of robotic uses and telemedicine options (43, 44). Robotics can be used in outbreaks of infections to minimize further exposure (45, 46): for disinfection, delivery of drugs and food, measuring vital signs, facilitating border control, and automatic disinfection (43). Telepresence robots allow for two-way communication and can be remotely controlled to provide support to those in isolation by connecting patients with family and physicians (35). Exposure to COVID-19 may stimulate further robotics research to address the risk of infectious diseases (42). Facilitating the integration of engineering, video technology, and infectious diseases specialists with government funding can have a notable impact on preventing future pandemics. A Smart Field Hospital trial in Wuhan, China, used robots to minimize COVID-19 exposure to patients and healthcare workers as robots and internet of things (IoT) devices provided medical services in the facility (47). The adoption of robots could be explored in infectious disease and crisis settings as a means to potentially improve health systems capacity and preparedness (47).

The Society of European Robotic Gynecological Surgery has released guidelines for robot-assisted surgery (RAS) and promoted the use of RAS over conventional laparoscopic and open surgery in managing infection risk (48). Where RAS is not possible, conventional laparoscopic is preferred over open surgery due to the reduced amount of physicians and PPE required, aerosol and bodily fluid risk, and shortened hospital stay (48, 49). The European Association of Urology similarly released COVID-19 guidelines, in which they pressed the need to manage smoke dispersion in robotic or laparoscopic surgery through the use of lowest intra-abdominal pressure and management of flow systems (50).

Potential issues associated with robotics-assisted telemedicine, include precision and interaction issues due to distance between patient and operator, network issues, and communication issues (49). Robot-assisted surgery has the potential to reduce COVID-19 exposure risk to medical professionals (51).

## Telemedicine Organizational Frameworks

**Figure 1:**
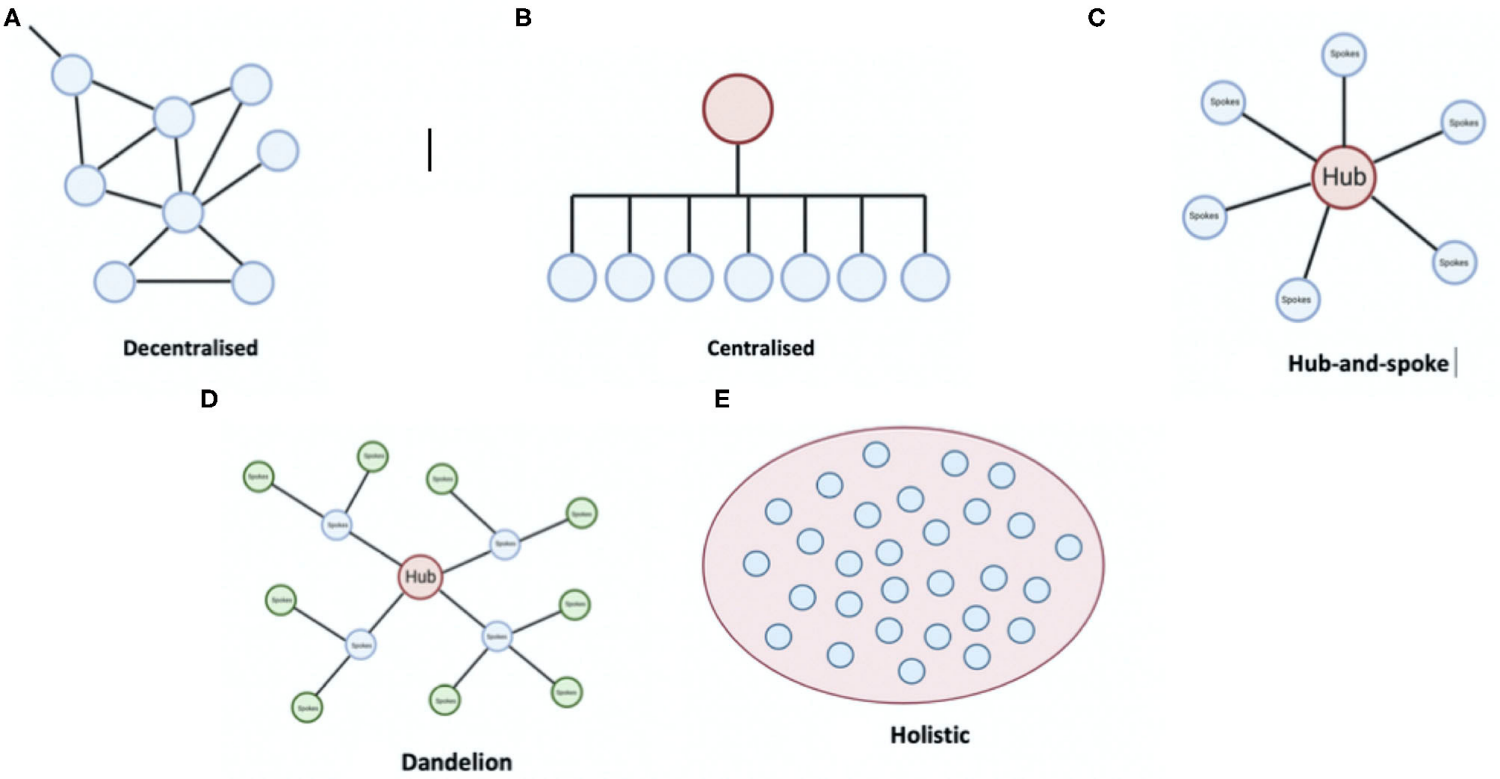
Traditional organizational frameworks such as **(A)** Decentralized, **(B)** Centralized, **(C)** Hub-and-spoke, **(D)** Dandelion, and **(E)** Holistic are shown.

Various traditional organizational frameworks are illustrated in Figure 1. One example of a traditional organization is the hub-and-spoke framework for stroke reperfusion therapy delivery (52), which is based on a framework of conventional and hierarchical positioning. It involves a centralized hub, which serves as a point of contact and instruction to several spoke sites that deliver care (53). This structure is suitable for the set-up and integration of telehealth networks but ultimately slows decision-making, depresses innovation, makes it difficult for spokes to communicate, and not everybody in the spoke has the capability of the hub (54). Due to cost-benefit and infrastructural reasons, as well as a shortage of neuro-interventionalists, not every spoke will have endovascular therapy capabilities. Additionally, barriers in access to health systems, applicable to culturally and linguistically diverse communities (CALD) as well as those from marginalized backgrounds or low resourced settings, merit special consideration (55, 56).

Centralized systems require strong leadership, otherwise, issues such as lack of efficiency, productivity, and physician well-being may develop (57). Decentralized systems can slow down the speed of uptake and pose inefficiency. Multiple hub-and-spokes are another framework that can be leveraged (58), e.g., in Africa, a capital city can have a hub overseeing the local state governments, which could further branch out. There also exists a holistic model, in which there is no hierarchy (59). Each element in the holistic structure is guided by the overarching vision of the organization.

## Discussion

Telemedicine offers a new modality of delivering medical & allied health services and communicating with patients (11). The current COVID-19 pandemic has catalyzed the uptake of telemedicine (12, 60). However, there are challenges concerning its geographical penetration, organizational structures, and infrastructure-related issues (12, 41). The consortium has outlined gaps in current telehealth approaches (11), including challenges to telemedicine implementation across geographics (12) and reliance on outdated organizational frameworks or operational structures discussed in the current report.

**Figure 2:**
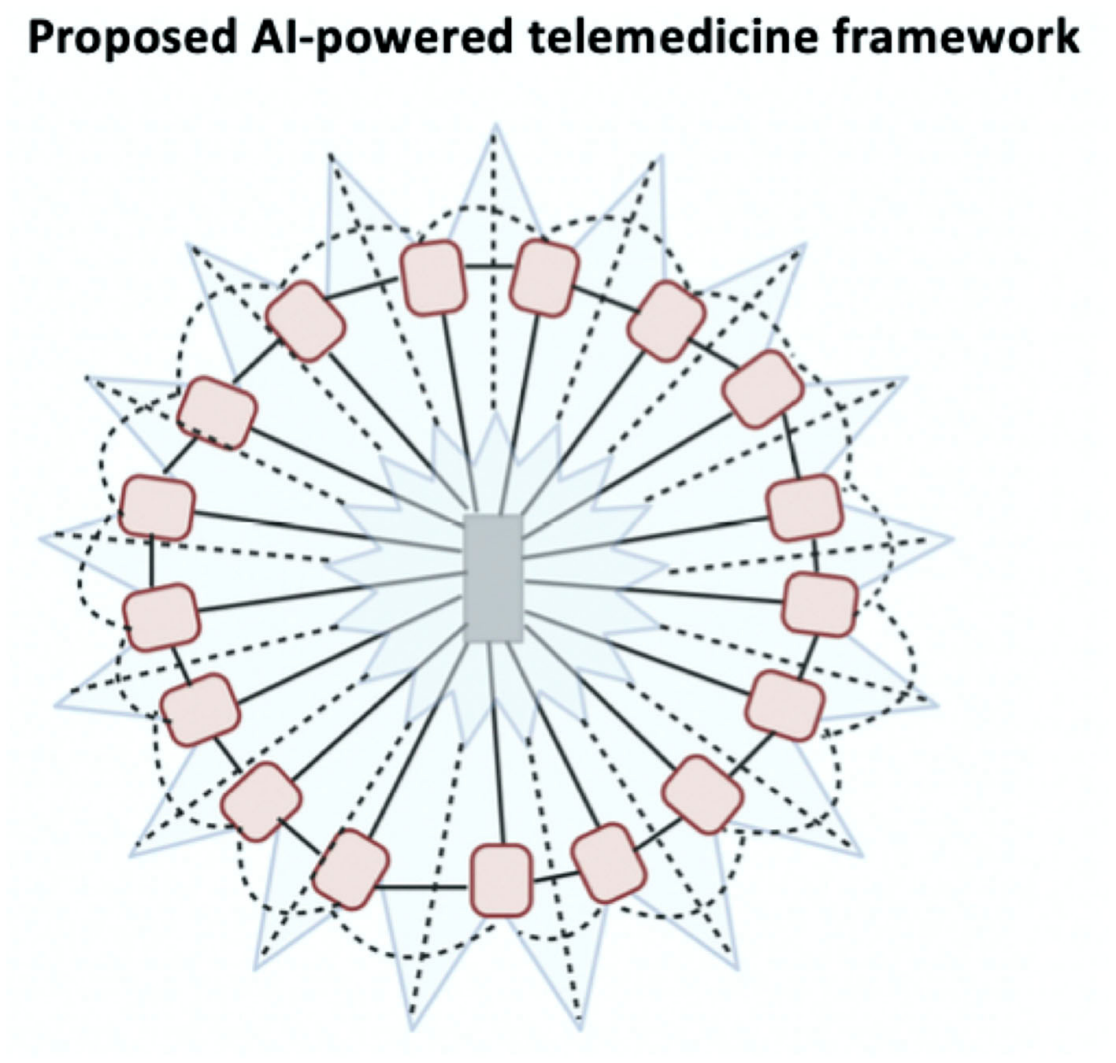
The proposed artificial intelligence assisted framework for telemedicine. This system centers around artificial intelligence (AI) engine that looks within the network to find the optimal resources within the geographical constraints and route incoming calls to the appropriate node (affiliated healthcare provider/facility). This self-evolving and innovative approach processing system would potentially allow increased telemedicine penetration while reducing the inefficiencies of the top-down or conventional organizational frameworks.

We propose a novel AI-powered staged-wise telemedicine organizational framework (61), which can potentially overcome the challenges associated with traditional organizational structures (Figure 2). Hub-and-spoke can be used as a foundational set-up to trigger the quick adoption of technology initially. However, after an initial period, once such a structure begins to stagnate and is no longer effective in skilling-up, a new organizational framework will replace the foundational model. The replacing model will consider leadership, technological, and organizational structure competence (61). This will involve a move toward performance and competency-based systems (62). For example, when an acute stroke call is made, an AI system will automatically find the best site of care depending on several variables, distance, availability of resources, clinicians' availability, and time constraints. This will focus on linking the patient to the best provider, thereby removing any elements of bureaucracy. An independent governance and ethical framework are necessary for oversight over the AI performance and any ethical issues (63, 64). An AI-powered system should prioritize the collective good and performance of the organization based on evidence-based management principles (65). It will be utilized to form linkages between providers. For instance, in the event of a cardiac emergency, the system could use the existing information on the patient to find the best teams to manage the patient. Links between a cardiologist and cardiac surgeon will be made so that they can communicate quickly. This involves role-based linkage based on questions such as who is available quickly, who is available in the region, and whether the practitioner agrees to treat such patients, rather than subjective person-to-person linkage. Ultimately, however, such an autonomous framework must also be able to assign responsibility in case of oversights. Hub-and-spoke systems allow teams to compete with each other and to curry favor from management. The proposed AI-assisted telemedicine organizational framework is focused on role-competence and linkages, rather than individuals or teams. We anticipate this would foster regular and continuous collaboration. Powered by big data and advanced data analytics dashboard, AI-powered systems can be insightful because they generate information about resource utilization in different regions, providing recommendations on system reorganization and clinician mobilization in a COVID-19 pandemic situation. This involves scoping the landscape and realigning across multiple levels to embed that particular intervention within the health systems, thereby promoting rapid research and innovation translation as well as integrating contributions based on local needs. Such a framework would imbibe resilience, innovation-driven technology, and scaled intelligence to enable systems to evolve and be responsive to local and emergent needs such as during an outbreak. COVID-19 has led to a sharp increase in the demand for telemedicine services (5, 34). It also has an impact on telemedicine providers, a sector that is facing unprecedented demand, which by some estimates has grown by 150% or more (66). For telemedicine to expand across geographics, it is necessary to account for geographical variations, cultural factors, and involvement of local stakeholders (12).

## Conclusion

To summarize, AI and robotics could play an important role in providing telemedicine services during an outbreak or public health emergency while limiting exposure to healthcare workers and health systems (14, 42, 43). The AI-assisted telemedicine framework proposed by the current consortium could be an enabler in improving telemedicine access and spread across medical specialties and geography. An international collaborative effort led by WHO, the current consortium, or similar organizations could pave a way to greater telemedicine penetration, especially to benefit the underprivileged and those living in the low-resourced settings (12).

## Author's Note

The COVID-19 pandemic is causing an unprecedented public health crisis impacting healthcare systems, healthcare workers, and communities. The COVID-19 Pandemic Health System REsilience PROGRAM (REPROGRAM) consortium is formed to champion the safety of healthcare workers, policy development, and advocacy for global pandemic preparedness and action.

## Author Contributions

SBh devised the project, the main conceptual ideas, including the proposal for a new AI-powered telemedicine workflow, the proof outline, coordinated the writing, editing of the manuscript, and wrote the first draft of the manuscript. All authors discussed the results and recommendations and contributed to the final manuscript.

## Conflict of Interest

SP is the Vice President of Immersive Medicine at Luxsonic technologies, a medical technology company specializing in virtual/augmented reality for medical education, collaboration, and training. The opinions expressed in this article are those of the authors and do not necessarily represent the decisions, official policy, or opinions of the affiliated institutions. The remaining authors declare that the research was conducted in the absence of any commercial or financial relationships that could be construed as a potential conflict of interest.
